# Red-on-Yellow Queen: Bio-Layer Interferometry Reveals Functional Diversity Within *Micrurus* Venoms and Toxin Resistance in Prey Species

**DOI:** 10.1007/s00239-024-10176-x

**Published:** 2024-05-30

**Authors:** Daniel Dashevsky, Richard J. Harris, Christina N. Zdenek, Melisa Benard-Valle, Alejandro Alagón, José A. Portes-Junior, Anita M. Tanaka-Azevedo, Kathleen F. Grego, Sávio S. Sant’Anna, Nathaniel Frank, Bryan G. Fry

**Affiliations:** 1 Australian National Insect Collection, Commonwealth Scientific and Industrial Research Organisation, Canberra, ACT 2601 Australia; 2 Venom Evolution Lab, School of the Environment, The University of Queensland, Saint Lucia, QLD 4072 Australia; 3 Australian Institute of Marine Science, Cape Cleveland, QLD 4810 Australia; 4 Celine Frere Group, School of the Environment, The University of Queensland, Saint Lucia, QLD 4072 Australia; 5 Department of Biotechnology and Biomedicine, Technical University of Denmark, DK-2800 Kongens Lyngby, Region Hovedstaden Denmark; 6 Departamento de Medicina Molecular y Bioprocesos, Instituto de Biotecnología, Universidad Nacional Autónoma de México, 62210 Cuernavaca, Morelos Mexico; 7 Laboratório de Coleções Zoológicas, Instituto Butantan, São Paulo, São Paulo 05503-900 Brazil; 8 Laboratório de Herpetologia, Instituto Butantan, São Paulo, São Paulo 05503-900 Brazil; 9 MToxins Venom Lab, 717 Oregon Street, Oshkosh, WI 54902 USA

**Keywords:** Coral snake, Elapid, Three-finger toxin, Mimotope, Arms race

## Abstract

Snakes in the family Elapidae largely produce venoms rich in three-finger toxins (3FTx) that bind to the $\upalpha _1$ subunit of nicotinic acetylcholine receptors (nAChRs), impeding ion channel activity. These neurotoxins immobilize the prey by disrupting muscle contraction. Coral snakes of the genus *Micrurus* are specialist predators who produce many 3FTx, making them an interesting system for examining the coevolution of these toxins and their targets in prey animals. We used a bio-layer interferometry technique to measure the binding interaction between 15 *Micrurus* venoms and 12 taxon-specific mimotopes designed to resemble the orthosteric binding region of the muscular nAChR subunit. We found that *Micrurus* venoms vary greatly in their potency on this assay and that this variation follows phylogenetic patterns rather than previously reported patterns of venom composition. The long-tailed *Micrurus* tend to have greater binding to nAChR orthosteric sites than their short-tailed relatives and we conclude this is the likely ancestral state. The repeated loss of this activity may be due to the evolution of 3FTx that bind to other regions of the nAChR. We also observed variations in the potency of the venoms depending on the taxon of the target mimotope. Rather than a pattern of prey-specificity, we found that mimotopes modeled after snake nAChRs are less susceptible to *Micrurus* venoms and that this resistance is partly due to a characteristic tryptophan$\rightarrow$serine mutation within the orthosteric site in all snake mimotopes. This resistance may be part of a Red Queen arms race between coral snakes and their prey.

**Supplementary Information:**

The online version contains supplementary material available at 10.1007/s00239-024-10176-x.

## Introduction

The genus *Micrurus* is part of the group of elapids known as the ‘true coral snakes’ along with *Micruroides* and *Sinomicrurus*. *Micrurus* is the most speciose genus of elapids and can be found from Argentina to the southern United States of America (Campbell and Lamar 2004). Genetic and morphological evidence clearly distinguishes between two main clades within *Micrurus*: short-tailed and long-tailed (Campbell and Lamar 2004; Jowers et al. 2019, 2023). In general, the short-tailed species are found in South America and possess a triadal color pattern (e.g., red-black-white-black-white-black-red) and tricolored tails, but include some bicolored species with bicolored tails as well. The long-tailed species are somewhat more variable; these mostly Central and North American species include bicolored, monadal (e.g., red-yellow-black-yellow-red), and triadal patterns with mostly bicolored tails (Campbell and Lamar 2004; Jowers et al. 2019, 2023). These clades are often referred to in the literature as ‘triadal’ and ‘monadal’ (Jowers et al. 2019, 2023; Gómez et al. 2021), but exceptions to these rules of color patterns abound. For the purposes of this paper, we will use the ‘short-tailed’ and ‘long-tailed.’ The venom composition of the two groups also differs somewhat in that the venoms from short-tailed species are usually (though not exclusively) dominated by three-finger toxins (3FTx). In contrast, long-tailed species produce venoms that range from being almost entirely composed of 3FTx to mostly phospholipase A_2_s (PLA_2_s) (Lomonte et al. 2016; Sanz et al. 2019).

One of the most widespread toxic activities of 3FTx is antagonistic binding to the orthosteric site (the acetylcholine binding region) of nicotinic acetylcholine receptor (nAChR) $\upalpha _1$ subunits (two of which along with a $\upbeta$, $\updelta$, and $\upepsilon$, form the heteropentameric muscular nAChR subtype (Tae and Adams 2023)), which are located at the neuromuscular junction (Galzi et al. 1991; Nirthanan and Gwee 2004). This disrupts muscle contraction which can cause flaccid paralysis; this mode of action is often referred to as $\upalpha$-neurotoxicity (Barber et al. 2013). The plesiotypic form of 3FTx—which can be found in venoms from many caenophidian snakes at varying abundances—contain 10 cysteine residues, which form 5 disulfide bonds to stabilize the structure of the toxin (Utkin et al. 2015). Ancestors of the family Elapidae evolved a derived form with only 8 cysteines; these ‘short-chain 3FTx’ lost the second and third cysteines—which together form a disulfide bridge in Loop I of the plesiotypic toxins—and are much more potent as a result (Fry et al. 2003). A further derived form known as ‘long-chain 3FTx’—also found only in elapid venoms—has evolved two new cysteines which form a disulfide bridge and stabilize Loop II (Utkin et al. 2015; Nirthanan and Gwee 2004). Although these long-chain toxins also contain 10 cysteines, the placement of cysteine residues is distinct from the plesiotypic form and protein sequence phylogenetic analyses confirm that they are derived from short-chain toxins (Fry et al. 2003; Utkin et al. 2015; Koludarov et al. 2023). Long-chain and short-chain neurotoxins are known to compete with each other when binding to the nAChR $\upalpha _1$ subunit, but they exhibit different specificities and kinetics (short-chain toxins bind to and dissociate from the nAChR much quicker) indicating slightly different biochemical mechanisms of action and target sites (Ackermann and Taylor 1997; Silva et al. 2018). Recent studies using cryogenic electron microscopy to investigate the binding of 3FTx to nAChRs confirm that plesiotypic, short-chain, and long-chain toxins all bind to the target receptors in noticeably different fashions (Rahman et al. 2020; Nys et al. 2022; Shenkarev et al. 2022).

Bio-layer interferometry (BLI) can be used as a method to assess the binding of $\upalpha$-neurotoxic snake venoms to mimotopes (small synthetic molecules which mimic an epitope) that are designed to resemble the nAChR orthosteric site from a wide range of taxa (Kamat and Rafique 2017; Zdenek et al. 2019; Harris et al. 2020a, b, c). Past studies have shown that similar mimotopes interact strongly with $\upalpha$-bungarotoxin (the best-studied long-chain neurotoxin), but this has not been explicitly demonstrated for short-chain neurotoxins (Bracci et al. 2001, 2002; Kasher et al. 2001; Katchalski-Katzir et al. 2003). Other studies investigated mimotopes of the orthosteric site as potential treatments to inhibit the $\upalpha$-neurotoxic activity of snake venoms by acting as decoys that keep the toxins from binding to the genuine receptors and found that the results were quite consistent for long-chain toxins, but unreliable for short-chain toxins (Albulescu et al. 2019; Kudryavtsev et al. 2020; Lynagh et al. 2020). In this study, we use BLI to investigate the patterns of $\upalpha$-neurotoxicity between species of *Micrurus* and across different targets.

The long-chain neurotoxins seem to have originally evolved shortly after the common ancestor of *Micrurus* and the more derived elapids diverged (Dashevsky and Fry 2018; Koludarov et al. 2023); 3FTx with the additional cysteines characteristic of long-chain neurotoxins have been published from all lineages of elapids besides *Calliophis* and the true coral snakes despite extensive transcriptomic investigations of these basal genera (Corrêa-Netto et al. 2011; Margres et al. 2013; Guerrero-Garzón et al. 2018; Tan et al. 2019; Bénard-Valle et al. 2020; Dashevsky et al. 2021; The UniProt Consortium 2023). Therefore, results obtained by studying *Micrurus* venoms more clearly demonstrate the effects of short-chain neurotoxins without the confounding presence of long-chain toxins. This makes these venoms an ideal test of whether and how short-chain neurotoxins bind to mimotopes of the nAChR orthosteric site. No other research has examined the binding of short-chain neurotoxins to $\upalpha _1$ subunit mimotopes specifically, and previous studies employing this method have only demonstrated significant binding from venoms that contain both short- and long-chain neurotoxins (Zdenek et al. 2019; Harris et al. 2020a, c).

Due to the high sensitivity of the BLI method, it can distinguish differences in how well a venom can bind to the different mimotopes, which can help assess the prey-specificity (greater toxicity against preferred prey species than other taxa) of that venom (Zdenek et al. 2019; Harris et al. 2020b, c). Prey specificity is a widespread phenomenon in snake venoms (da Silva and Aird 2001; Mackessy et al. 2006; Pawlak et al. 2006, 2008; Barlow et al. 2009; Heyborne and Mackessy 2013; Modahl et al. 2018; Sousa et al. 2018) and has been documented in *Micrurus* venoms using *in vitro* tests of whole venom (da Silva and Aird 2001). By using this BLI assay our results will clarify if this specificity is achieved through variation in the ability of these venoms to bind to the orthosteric site of the nAChR in their prey.

However, if coevolution can drive the evolution of prey-selective venom, then it must also be considered that there is an opposing evolutionary pressure for the prey of *Micrurus* to become less susceptible to the toxins of their predators. Following the life-dinner principle, the selection pressure on prey populations may be even greater because the consequence of a predation attempt for them is death rather than a missed meal (Dawkins and Krebs 1979). Resistance and reduced susceptibility to snake $\upalpha$-neurotoxins has been reported from a number of taxa which are either predators or prey of venomous snakes (Holding et al. 2016; Khan et al. 2020). Mongooses, pigs, and hedgehogs—which are known to feed on venomous snakes—are less susceptible to elapid $\upalpha$-neurotoxins due to convergent amino acid substitutions in the orthosteric binding site of the muscular nAChR subunit and similar mutations can be found in elapids themselves (Barchan et al. 1992, 1995; Takacs et al. 2001, 2004; Drabeck et al. 2015). Primates have also been shown to possess nAChR mutations which reduce their susceptibility to $\upalpha$-neurotoxins (Harris et al. 2021). Whether the immunity in elapids evolved as auto-resistance, in response to intraguild predation, or both, is unclear. Some lizards and other snakes also show reduced susceptibility to the effects of both long-chain and short-chain neurotoxins (Burden et al. 2006). Further, a study addressing the effects of *M. nigrocinctus* venom on different taxa found that cows are less susceptible than horses (Bolaños et al. 1975). Another demonstrated a marked difference between two genera of dipsadine snakes that are known prey of *M. nigrocinctus*: *Geophis* spp. were susceptible while *Ninia* spp. were more resistant (Urdaneta et al. 2004). The latter study incubated *M. nigrocinctus* venom with *N. maculata* blood serum which had a protective effect when injected into mice; this suggests that the mechanism of resistance is that the serum of *N. maculata* is able to directly inhibit the toxins rather than the presence of mutations to their muscular nAChR subunit allowing them to resist the toxic effects (Urdaneta et al. 2004). A more recent experiment using the venom of *M. tener* found that the snake species *Conopsis lineata* could survive higher venom doses than mice, but that both groups were paralyzed at the same dosage (Bénard-Valle et al. 2014).

Based on the evidence that some *Micrurus* venoms exhibit prey-selectivity (da Silva and Aird 2001) and some natural prey might have a reduced susceptibility to their venom (Urdaneta et al. 2004), we wanted to test the effects of both short-tailed (8 venoms) and long-tailed (7 venoms) *Micrurus* venoms using BLI. We analyzed these venoms using 12 different mimotopes designed to resemble the muscular nAChR orthosteric site from a range of taxa, including some that are natural prey of *Micrurus* and others that are not. We also wanted to assess if the serine (S) at position 187 of the muscular nAChR (the orthosteric site runs from 187–200 in the overall sequence of the receptor subunit) found in most snake lineages conferred any resistance/ reduced susceptibility since this is a significant biochemical change from the ancestral tryptophan (W) (Khan et al. 2020). To further test this, we designed a mutant based on the blind snake mimotope, substituting the natural 187 S for the ancestral W. Taken together, these data provide insights into the evolution of venom function within *Micrurus* and their coevolution with prey taxa.

## Results and Discussion

**Fig. 1:**
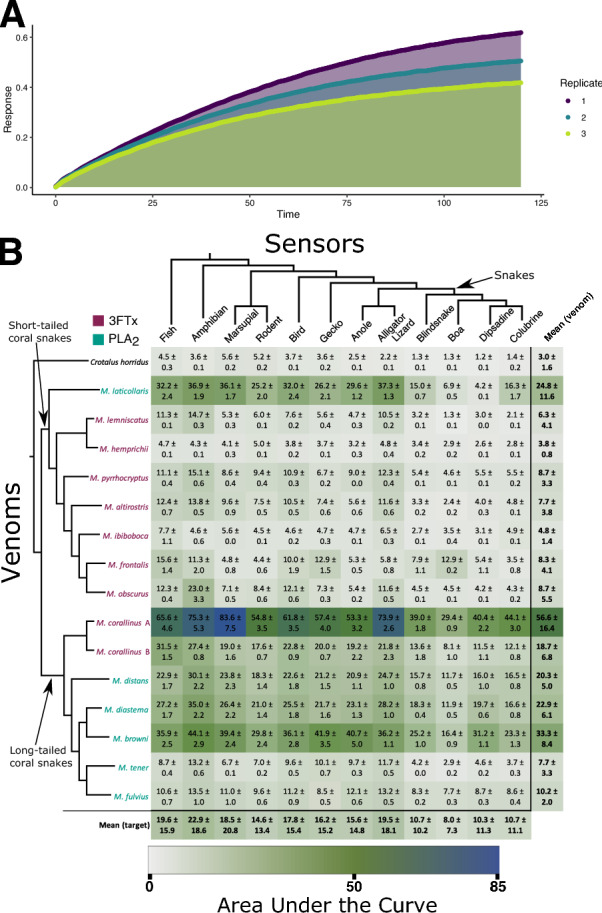
The binding of *Micrurus* toxins to muscular nAChR mimotopes varies greatly depending on the individual venom as well as the target: **A** Example results of *Micrurus corallinus* A venom binding to Dipsadine mimotope. Shaded regions indicate the area under the curve for each replicate that is then averaged. **B** Cells show the area under the curve (Mean ± Standard Deviation, $N = 3$) for the association step of the binding of each venom to each mimotope. Phylogeny on the left displays relationships between the venoms tested (short-tailed and long-tailed clades indicated by arrows, 3FTx-heavy venoms in purple and PLA_2_-heavy venoms in teal), while the one on top displays the relationship between organisms on which mimotopes were modeled (clade of mimotopes based on snake sequences indicated with arrow). *Crotalus horridus* venom was used as a negative control as it contains many related protein families, but none which target the nAChR (Rokyta et al. 2013, 2015). Phylogenetic topology for *Micrurus* species was primarily adapted from the results of two recent phylogenies (Jowers et al. 2023; Reyes-Velasco et al. 2020) and checked for consistency with previous findings (Slowinski 1995; Gutberlet Jr and Harvey 2004; Pyron et al. 2013; Figueroa et al. 2016; Lee et al. 2016; Lomonte et al. 2016; Zheng and Wiens 2016; Zaher et al. 2019; Jowers et al. 2019; Gómez et al. 2021)

Consistent with previous studies on neurotoxic snake venoms, the BLI method was able to detect orthosteric binding by *Micrurus* venoms, and interesting patterns emerged both in terms of the differences within *Micrurus* and between the targets (Fig. 1). Our results clearly show that the strength of binding measured by our assay depended on the species of origin for both the venom and mimotope. While short-tailed *Micrurus* venoms elicited very weak or no binding at all compared to the control of *Crotalus horridus* (a species chosen for its lack of nAChR-binding neurotoxins (Rokyta et al. 2013, 2015; Harris et al. 2020b), the venoms of many long-tailed species showed considerable binding to the targets (Fig. 1). Since *Micrurus* venoms do not contain long-chain neurotoxins, this confirms that the assay can be used to analyze the activity of short-chain neurotoxins that bind to the orthosteric site of the nAChR. A previous study that investigated the interaction of short-chain neurotoxins and nAChR mimotopes had reported very little binding, but those mimotopes were designed to resemble the orthosteric binding region of the $\upalpha _7$ subunit (Albulescu et al. 2019), which is not a physiologically relevant target since it is located within the central nervous system, rather than at the neuromuscular junction (Gotti and Clementi 2004). 3FTx circulating in the bloodstream or lymphatic system are too large to reach the central nervous system but can readily affect the neuromuscular junction, which is why $\upalpha$-neurotoxins have evolved to immobilize prey by inhibiting this more accessible physiological target. Short-chain neurotoxins are known to bind almost exclusively to the $\upalpha _1$ rather than the $\upalpha _7$ subunit (Ackermann and Taylor 1997; Servent et al. 1997). Because the mimotopes in this study were designed to resemble the $\upalpha _1$ subunit, this easily explains why our assay could measure the effects of these toxins where previous research had not. While this might be taken as evidence that decoy peptides that resemble the $\upalpha _1$ subunit could have even greater potential as a therapeutic tool to help mitigate the neurotoxic symptoms of bites from coral snakes than the $\upalpha _7$-derived ones (Albulescu et al. 2019), other patterns in our results cut against this conclusion.

One of the striking trends in our data was the variability within and between species of *Micrurus*. The venom with the largest effect on average (and for every individual mimotope) was a sample of *M. corallinus*, while the other individual of this species that we tested had only the 6th highest average effect. This discrepancy between the two samples could result from regional variation within this species or could be an indicator of potential cryptic speciation in this lineage. While there is no direct evidence for this latter hypothesis, species complexes are so widespread in this incredibly speciose genus (Roze 1996; Campbell and Lamar 2004; Terribile et al. 2018; Nascimento et al. 2019) and new species which bear strong external similarity to their congeners are described with enough frequency (Di-Bernardo et al. 2007; Pires et al. 2014; da Silva et al. 2015; Bernarde et al. 2018) that the possibility bears mentioning. There is also a phylogenetic trend: the long-tailed *Micrurus* elicited a much greater response than their short-tailed relatives. Several short-tailed *Micrurus* venoms did not show appreciably stronger binding than our negative control (*Crotalus horridus*) venom. This is especially interesting in light of previous findings that the venoms produced by some long-tailed species (such as *M. diastema*, *M. browni*, *M. tener*, and *M. fulvius*) are dominated by PLA_2_s—which are not known to bind to nAChRs—rather than 3FTx (Lomonte et al. 2016). Due to the variability of each venom’s binding across the different mimotopes, we could not simply compare the means for each venom. Instead, we created a factor denoting whether the predominant toxin family in a given venoms was 3FTxs or PLA_2_s and used two-way unbalanced ANOVA with type III sum of squares to simultaneously test the effects of this factor, the mimotope, and their interaction. This analysis did not find a significant effect for the dominant toxin family ($\textit{p} =0.12$) or the interaction between toxin family and sensor ($\textit{p} =0.99$), but the sensor alone did have a significant effect ($\textit{p} =0.004$). We then repeated this analysis using a factor capturing the short- and long-tailed clades rather than the dominant toxin family. We found that the average difference between short-tailed and long-tailed species was greater than twofold for each mimotope, that this was a statistically significant trend ($\textit{p} <0.001$), that the variation across mimotopes was also significant ($\textit{p} =0.02$), but the interaction between the two was not ($\textit{p} =0.71$).

Outliers to this general pattern include the idiosyncratic species *M. laticollaris* at the base of the short-tailed clade, which was comparable to most long-tailed venoms, and the two North American species (*M. tener* and *M. fulvius*) which had much less effect than other long-tailed species. The similarity of *M. laticollaris* to long-tailed species is especially surprising because the sequence of its primary $\upalpha$-neurotoxin—MlatA1, UniProt K9MCH1 (Carbajal-Saucedo et al. 2013; Guerrero-Garzón et al. 2018)—is most similar to those of other short-tailed species: the top 10 most similar sequences in UniProt from other species all belong to short-tailed rather than long-tailed *Micrurus* (The UniProt Consortium 2023). This activity shift could potentially be an evolutionary innovation related to the large clades of short-chain 3FTx that are unique to long-tailed *Micrurus* and display strong signals of positive selection (Dashevsky and Fry 2018). However, the relatively similar results between *M. laticollaris* and most of the long-tailed species suggest an alternate hypothesis: that high binding may instead be an ancestral trait and that the main branch of the short-tailed phylogeny evolved away from toxins that bind to the orthosteric site. This scenario also accords with the overall evolutionary history of the 3FTx since the ancestral state of the family—represented by the plesiotypic toxins—is to bind to the orthosteric site, and this activity has been widely demonstrated for more derived toxins as well (Barber et al. 2013; Utkin et al. 2015; Rahman et al. 2020; Nys et al. 2022; Shenkarev et al. 2022).

This does not imply that species which produced small effects in this assay are necessarily less dangerous. Indeed, several of these species have been responsible for fatal bites in the past (Norris et al. 2009; Otero-Patiño 2014; Bucaretchi et al. 2016). This does not even necessarily mean that venoms which produce small effects do not inhibit the nAChR: post-synaptic neurotoxicity has been demonstrated in the venoms from a range of short-tailed (*M. dissoleucus* (Renjifo et al. 2012), *M. laticollaris* (Carbajal-Saucedo et al. 2013, 2014), *M. lemniscatus* (Cecchini et al. 2005; Floriano et al. 2019), *M. mipartitus* (Renjifo et al. 2012), *M. obscurus* (Yang et al. 2017), *M. pyrrhocryptus* (Yang et al. 2017), and *M. surinamensis* (Harris et al. 2020a)) and long-tailed (*M. dumerilii* (Serafim et al. 2002), *M. fulvius* (Snyder et al. 1973; Yang et al. 2017; Foo et al. 2019), and *M. tener* (Yang et al. 2017)) species. Earlier work has suggested that short-chain toxins might act as allosteric inhibitors of the nAChR: toxins which bind to a site other than the orthosteric site but still disrupt the receptor’s normal function (Nirthanan 2020; Harris et al. 2020a). This site may be located at other regions of the $\upalpha _1$ subunit or other subunits of the muscular nAChR (which also include $\upbeta$, $\updelta$, and $\upepsilon$ subunits). This previous research was focused on aquatic elapids including *M. surinamensis*, which is a short-tailed species, and our results suggest that allosteric 3FTx may be a feature in most of the rest of the clade as well. It has been put forward that allosteric 3FTx may be able to incapacitate prey faster than the more common orthosteric toxins, but would likely not bind as strongly (Barber et al. 2013; Harris 2022).

The possibility of wide spread allosteric toxins in *Micrurus* venoms casts doubts on the potential efficacy of decoy peptides as a potential therapeutic to combat bites from the genus. Some more recent studies into this area have used decoy peptides that do resemble the orthosteric site of the muscular nAChR and showed very potent binding with long-chain toxins (Kudryavtsev et al. 2020; Lynagh et al. 2020), but if there are short chain toxins that are able to cause severe neurotoxicity without significant affinity for the orthosteric site, then such peptides are unlikely to be of much use in combatting their effects. Even beyond the possibility of allosteric 3FTx, *Micrurus* venoms have been shown to contain potent neurotoxins from other toxin families that act through different mechanisms, including targeting the presynaptic side of the neuromuscular junction (Dal Belo et al. 2005; Oliveira et al. 2008; Bohlen et al. 2011; Terra et al. 2015; Floriano et al. 2019; Santos et al. 2020). This is particularly relevant for species that are known to produce primarily PLA_2_s—which are often presynaptically neurotoxic—in their venom rather than 3FTx, such as some populations of *M. laticollaris*, *M. lemniscatus*, *M. ibiboboca*, *M. diastema*, *M. browni*, *M. tener*, and *M. fulvius* (Lomonte et al. 2016; Sanz et al. 2019). Since the BLI method can only assay binding to the orthosteric site, toxins that act on other sites, be they elsewhere on the receptor or on the other side of the neuromuscular synapse, will not display any binding in our results.

**Fig. 2:**
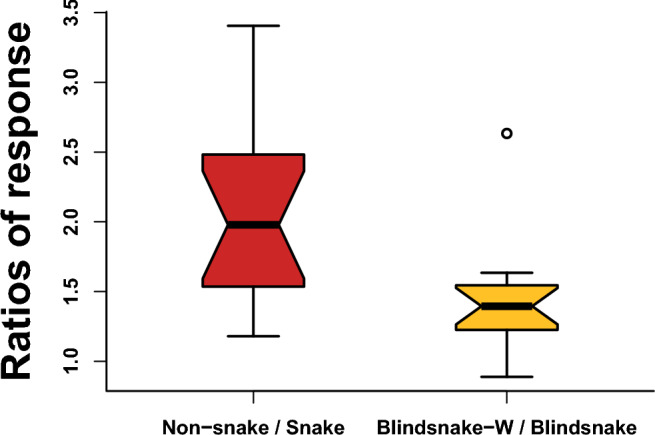
Snake nAChR are consistently less vulnerable to $\upalpha$-neurotoxins, partially due to the W187S mutation: response of non-snake mimotopes to each *Micrurus* venom are on average higher than the response of snake mimotopes to that same venom (indicated by the ratios greater than 1), similarly the pseudo-ancestral blind snake mimotope without the W187S mutation are more vulnerable than the normal blind snake mimotope

Another interesting facet of our results is the fact that mimotopes derived from taxa other than snakes tend to be more sensitive to *Micrurus* venoms (non-snake mimotopes were almost twice as susceptible as snake mimotopes on average, Fig. 2). To analyze these data, we used a similar approach as when we compared short-tailed and long-tailed venoms, but used a new factor based on whether the mimotope was derived from the nAChR sequence of a non-snake or a snake species and then analyzed this factor, the species of origin for the venom, and their interaction. This once again confirmed that there is significant variation between the potency of the venom from different species ($\textit{p} <0.001$), but also that non-snake mimotopes are more easily bound by *Micrurus* venoms than those derived from snakes ($\textit{p} <0.001$), and these variables interact significantly as well ($\textit{p} <0.001$). Given the high proportion of snakes in the diets of most *Micrurus* species (Jackson and Franz 1981; Roze 1996; Marques and Sazima 1997; Marques 2002; Ávila et al. 2010; da Silva Banci 2017), our hypothesis of prey-specificity driving venom evolution would generally predict increased potency against mimotopes derived from snake sequences. As discussed earlier, our assay is a window into a specific fraction of the total activity of a snake’s venom. It is possible that the holistic potency of these venoms would be greater against snake rather than non-snake targets, but within the limitation of our results we find evidence for prey resistance.

To further investigate the resistance of snake mimotopes, we created a pseudo-ancestral mimotope by modifying the sequence of the most basal snake mimotope (blind snake) to revert the derived snake serine (S) at position 187 back to the tryptophan (W) that most other taxa possess at that position. Two-way ANOVA shows that *Micrurus* venoms more easily bind to the pseudo-ancestral blind snake mimotope than the normal blind snake mimotope ($\textit{p} <0.001$). Variation between venoms from different species of *Micrurus* remained significant ($\textit{p} < 2 \times 10^{-16}$) and the interaction between the two variables was marginally significant ($\textit{p} =0.04$). While these results clearly indicate that the W187S mutation reduces susceptibility to the binding of *Micrurus* toxins, Welch’s two-sample t-test shows that the ratio of the average response to a given venom of non-snake mimotopes to that of snake mimotopes ($2.01\ \pm \ 0.65$) is significantly greater than that the ratio between the pseudo-ancestral blind snake mimotope and the normal one ($1.43\ \pm \ 0.40$, $p =0.007$, Fig. 2). The source of this increased resistance is somewhat enigmatic since no other derived mutations are found in all and only snake sequences. Interestingly, the resistance conferred by this mutation seems to operate through biochemical mechanisms that do not interfere with the greater potency of the toxins from long-tailed species. The long-tailed species have similar response ratios between non-snake and snake mimotopes as the short-tailed species.

While 3FTx are an ancient family of snake toxins and have even been reported from very basal snake lineages including Pythonidae and Cylindrophiidae (Fry et al. 2013; Hargreaves et al. 2014), there is no published evidence they evolved before the divergence of the thread snakes and blind snakes from the more derived Alethinophidian snakes (Dashevsky et al. 2018; Koludarov et al. 2023). If indeed $\upalpha$-neurotoxic 3FTx had not evolved before the most recent common ancestor of all extant snakes, selection to resist the binding of these toxins cannot explain the widespread occurrence of the W187S mutation. This tryptophan residue is largely conserved in most other chordate taxa and negative selection has been shown to act on this site in most lineages (Khan et al. 2020). A similar scenario has been observed in lineages that can resist toad toxins, but related taxa in toad-free environments tend to lose the adaptation (Ujvari et al. 2015), suggesting the adaptation comes at a physiological cost and is lost when it is no longer offset by the benefit of resistance. It is unclear whether the W187S mutation arose as a deleterious trait and persisted through chance, if it was a neutral mutation because something about the biology of snakes negated the penalty that other taxa would face, or if the common ancestor of snakes was exposed to some selective pressure that made the mutation adaptive at the time. Since then, however, snakes whose venoms contain 3FTx—many of which are ophiophagus—have become widespread. Whatever its origins, the W187S mutation certainly seems to have been exapted to help protect snakes armed with these neurotoxins from the risk of self-envenomation and, perhaps more importantly, to protect snakes broadly from predation by their neurotoxic relatives.

Another factor that is not captured by our results is the possibility of prey taxa producing venom inhibitors rather than altering the targets, as has been well characterized for other families of snake toxins (Holding et al. 2016). Indeed, preliminary evidence suggests this might be the case for some snakes (Bolaños et al. 1975; Rodrigues et al. 2020, 2021). Coupled with the W187S mutation at the muscular nAChR orthosteric site, such venom inhibitors could have a synergistic effect and grant high levels of resistance to $\upalpha$-neurotoxicity. Along with other factors, including relaxed selective constraint and pressure from predators, multi-faceted resistance has been suggested as one potential reason why so many neurotoxic snake venoms seem to be toxic to the point of overkill (Broad et al. 1979; Mebs 2001; Kundu et al. 2015; Aird et al. 2017; Gangur et al. 2018; Healy et al. 2019). If their prey (other snakes) are resistant then venomous snakes might need to evolve toward larger doses and/or more toxic venoms due to Red Queen dynamics (Van Valen 1973). Since the model organisms (usually mice) are not participants in this specific arms race, the tests carried out on them may wrongfully suggest the venoms in question are much more toxic than they are in practice in their proper ecological context. With this in mind, the reduced susceptibility to $\upalpha$-neurotoxins we observed among snakes relative to non-snake taxa may be just part of an ongoing arms race between predator and prey. Two studies have been carried out comparing the effects of *Micrurus* venoms on dipsadine snake species and lab mice and both suggest that these snakes are much less susceptible to the lethal effects of the venoms, with the least resistant snake species exhibiting LD_50_s threefold greater than the mice (Urdaneta et al. 2004; Bénard-Valle et al. 2014). However, there is substantial variation even within this subfamily of snakes and the more recent study found that there was no difference in the dosage required to paralyze the mice and snakes, which is a more ecologically relevant measure when considering the predatory purpose of the venom (Bénard-Valle et al. 2014).

## Conclusion

This study examines a very particular mode of action among *Micrurus* venoms—binding to the orthosteric site of the nAChR—and reveals two particularly interesting patterns. The first is that there is substantial variation between and within *Micrurus* species and that the phylogenetic pattern suggests that the ancestral state of the genus was likely to produce toxins which bind strongly to the orthosteric site. Several lineages—including most of the short-tailed clade as well as the two North American species in the long-tailed clade—have shifted away from this specific target. The second major trend was that targets which mimicked snake sequences were less susceptible to *Micrurus* venoms than those which mimicked other taxa. This is due, in part, to a W$\rightarrow$S mutation in the orthosteric region, which is shared by snakes, but rare elsewhere. However, there is much more to even a simple venom than its potency at one specific site of one physiological target and these results may not be representative of the broader picture of the arms race between $\upalpha$-neurotoxic elapids and their snake prey.

## Methods

### Venom Samples

All venoms were stored as lyophilized powder as part the long-term cryogenic collections in the Venom Evolution Lab. Venoms for *Micrurus fulvius* (FL, USA), *M. obscurus* (Brazil), and *M. tener* (TX, USA) were supplied by Miami Serpentarium. Nathaniel Frank of MToxins Venom Lab provided *M. corallinus* A venom. Venom from *M. pyrrhocryptus* was from an individual in the captive collection of Iwan Hendrikx that originally came from Suriname. José A. Portes-Junior, Anita M. Tanaka-Azevedo, Kathleen F. Grego, and Sávio S. Sant’Anna of Instituto Butantan provided the majority of the Brazilian venoms: *M. altirostris* (pooled from 3 individuals), *M. corallinus* B (pooled from 3 individuals), *M. frontalis*, *M. hemprichii*, *M. ibiboboca* (pooled from 2 individuals), and *M. lemniscatus*. Venoms of Mexican *Micrurus* from Alejandro Alagón—*M. browni*, *M. diastema*, *M. distans*, and *M. laticollaris*—were manually extracted from *Micrurus* specimens kept at the “Herpetario Cantil” from Instituto de Biotecnología–Universidad Nacional Autónoma de México. After extraction, venoms were diluted in MilliQ water, centrifuged 3 min at 10,000 RCF to remove insoluble material, lyophilized and stored at 4 °C until shipment.

As no live animals were used for this study, and all venoms were from previously collected stocks, no animal ethics approvals were required for this work. These lyophilized venoms were resuspended in water, centrifuged (4 °C, 5 min at 14,000 RCF), and diluted into a solution of 1 $\frac {\textrm{mg}}{\textrm{ml}}$ of venom in a 1:1 mixture of water and glycerol. Protein concentrations were measured using a NanoDrop 2000 UV–Vis Spectrophotometer (Thermofisher, Sydney, NSW, Australia).

### Mimotope Production and Preparation

The sequences for the muscular nAChR were downloaded from the following UniProt entries: *Tetronarce californica* (fish, P02710), *Xenopus tropicalis* (amphibian, F6RLA9), *Sarcophilus harrisii* (marsupial, G3W0J0), *Rattus norvegicus* (rodent, P25108), *Gallus gallus* (bird, E1BT92), *Gekko japonicus* (gecko, GenBank: XM015426640), and *Anolis carolinensis* (anole, H9GA55), *Barisia imbricata* (alligator lizard, A0A859JD35), *Anilios bituberculatus* (blind snake, UniProt: A0A7L5PIU6), *Boa constrictor* (boa, A0A7L5PLV8), *Oxyrhopus rhombifer* (Dipsadine, A0A7L5PLX4), and *Pantherophis spiloides* (Colubrine, A0A7L5PL14). These sequences were then aligned using AliView 1.18 (Larsson 2014) and were trimmed down to the 14 amino acids of the orthosteric binding site. The alignment of these sites can be found in the Supplementary Data S1.

Subsequently, following previous protocols (Chiappinelli et al. 1996; Bracci et al. 2001; Kasher et al. 2001; Bracci et al. 2002) mimotopes of these sequences were developed by GenicBio Ltd (Shanghai, China). As per previous studies (Bracci et al. 2001), the cysteine doublet in the orthosteric binding site sequence was replaced in peptide synthesis steps with serine doublet to avoid uncontrolled postsynthetic thiol oxidation. Research has shown this has no effect on the analyte-ligand complex formation (Tzartos and Remoundos 1990; McLane et al. 1991, 1994). The mimotope was further synthesized to a biotin linker bound to two aminohexanoic acid (Ahx) spacers to form a 30 Å linker between biotin and the peptide. This provides conformational freedom for the analyte-ligand complex. The purpose of adding the biotin-Ahx complex is to allow the biotin molecule to bind non-covalently to the avidin pocket of the streptavidin-coated disposable biosensors used in the bio-layer interferometry assay. Dried stocks of synthesized mimotopes were solubilized in 100% dimethyl sulfoxide (DMSO) and then diluted 1:10 in deionized water to make a final working stock concentration of 50 $\frac{{\upmu }{\textrm{g}}}{\textrm{ml}}$ and stored at −80 ^∘^C until use.

### Bio-Layer Interferometry

Full details of the assay, including a full methodology and data analysis, can be found in the validation study (Zdenek et al. 2019) and further publications using this protocol (Harris et al. 2020a, b, c, 2021; Chowdhury et al. 2022). In summary, the BLI assay was performed on the Octet Red 96 system (ForteBio, Fremont, CA, USA). Venom (analyte) samples were diluted at 1:20 (a final experimental concentration of 50 $\frac{{\upmu }{\textrm{g}}}{\textrm{ml}}$ per well). Mimotope aliquots were diluted at 1:50 (a final concentration of 1 $\frac{{\upmu }{\textrm{g}}}{\textrm{ml}}$ per well). The assay running buffer was 1X DPBS with 0.1% BSA and 0.05% Tween-20. Before experimentation, streptavidin biosensors were hydrated in the running buffer for 30–60 min. Elimination of bound venom toxins (regeneration) was performed using a standard acidic solution glycine buffer (10 mM glycine (pH 1.5$-$1.7) in ddH2O). Raw data are provided in Supplementary Data S2 and line charts similar to Fig. 1A can be found in Supplementary Figs. S1–S12. All data obtained from BLI on Octet Red 96 system (ForteBio) were processed following the protocol in the validation study for this assay (Zdenek et al. 2019). In brief, the raw data were exported to a.csv file. Area under the curve for the association step of each samples was estimated using trapezoidal approximation in Microsoft Excel 15.28. Because venoms are heterologous mixtures of multiple different toxins of unknown molarity in the assay we were unable to estimate the on and off rates of the binding reactions necessary to calculate dissociation constants. The presence of multiple toxins, some of which were able to weakly associate with the mimotopes also complicated the dissociation phase of the reaction which is why we focused on the association phase. Further statistical analyses were carried out in the RStudio 2023.06.1+524 implementation of R 4.3.1 (RStudio Team 2015; R Core Team 2015). These data can be found in the Supplementary Material.

### Supplementary Information

Below is the link to the electronic supplementary material.

Supplementary material 1 (FASTA 0.4 kb)

Supplementary material 2 (CSV 4026.7 kb)

Supplementary material 3 (PDF 17727.8 kb)
