# Supplementary material for: Red-on-Yellow Queen: Bio-Layer Interferometry Reveals Functional Diversity Within Micrurus Venoms and Toxin Resistance in Prey Species
Source: J Mol Evol. 2024 May 30;92(3):317–28. doi: 10.1007/s00239-024-10176-x (PMC11168994; doi:10.1007/s00239-024-10176-x)
Supplement: Supplementary file 3 — Supplementary material 3 (PDF 17727.8 kb) [file 239_2024_10176_MOESM3_ESM.pdf]

Red-on-yellow queen: Supplementary figures

D Dashevsky, RJ Harris, CN Zdenek, M Bénard-Valle, A Alagón,
JA Portes-Junior, N Frank, BG Fry

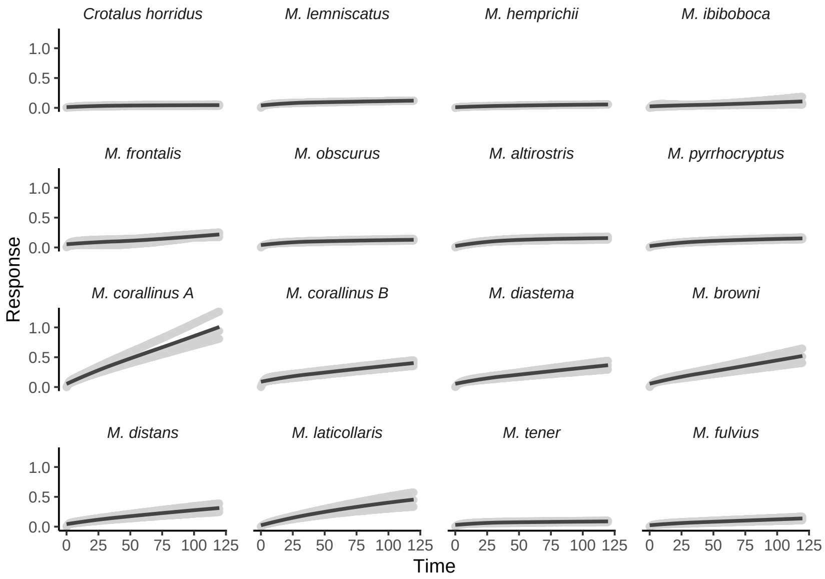

Figure S1: Bio-layer interferometry results for *Micrurus* venoms binding to a mimotope based on fish (*Tetronarce californica*, UniProt P02710) α_1-nAChR sequence. Individual datapoints in grey with loess-smoothed average in black.

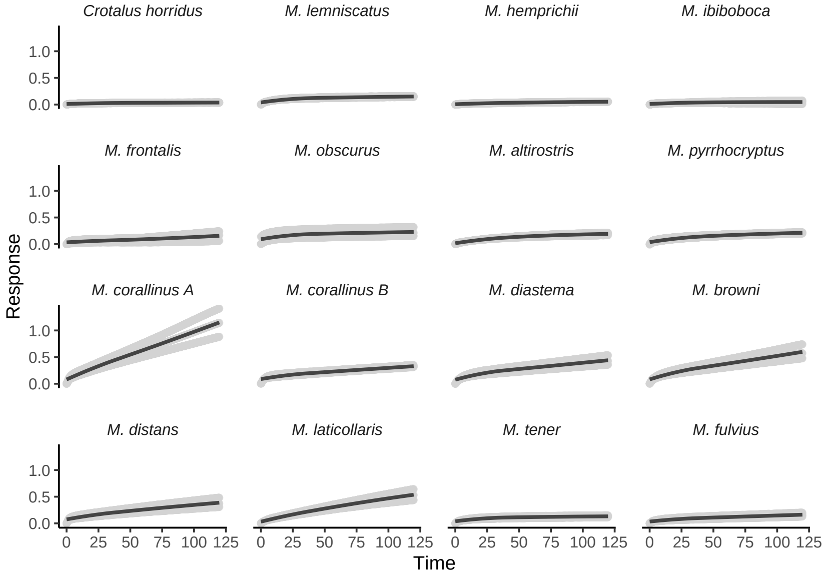

Figure S2: Bio-layer interferometry results for *Micrurus* venoms binding to a mimotope based on amphibian (*Xenopus tropicalis*, UniProt F6RLA9) α_1-nAChR sequence. Individual datapoints in grey with loess-smoothed average in black.

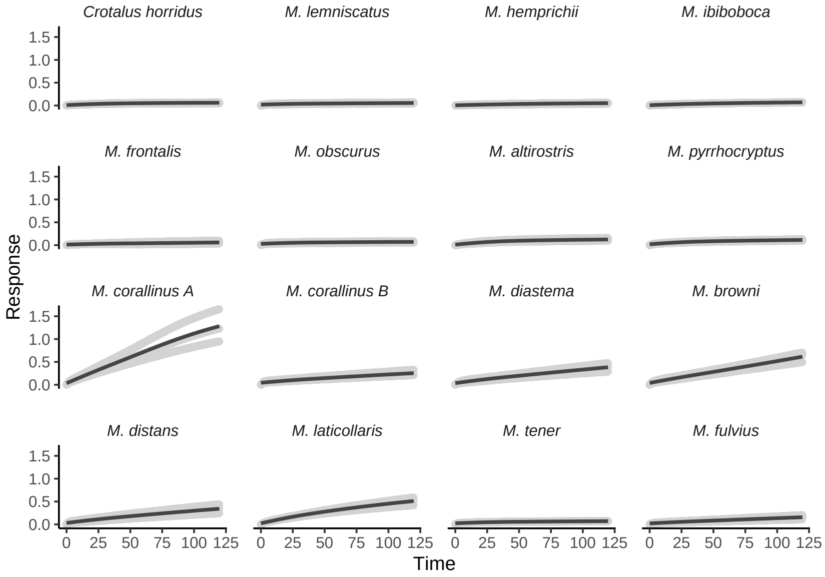

Figure S3: Bio-layer interferometry results for *Micrurus* venoms binding to a mimotope based on marsupial (*Sarcophilus harrisii*, UniProt G3W0J0) α_1-nAChR sequence. Individual datapoints in grey with loess-smoothed average in black.

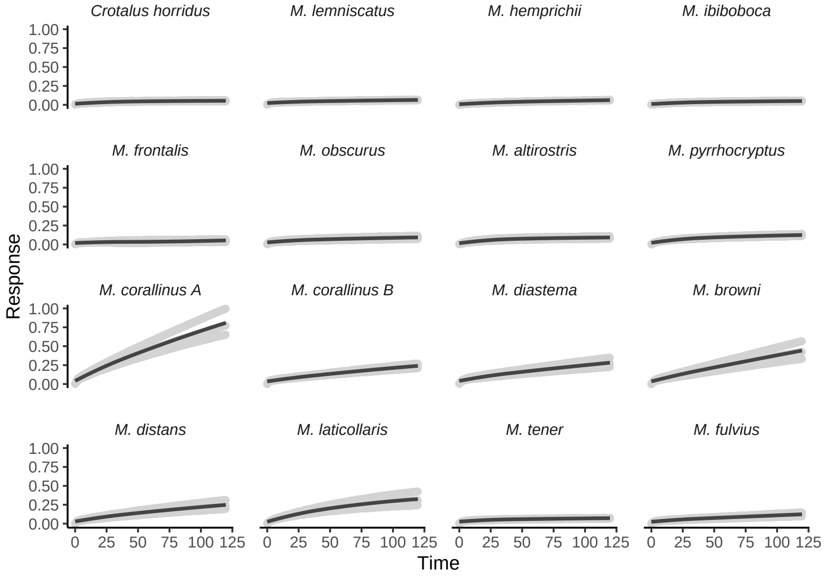

Figure S4: Bio-layer interferometry results for *Micrurus* venoms binding to a mimotope based on rodent (*Rattus norvegicus*, UniProt P25108) α_1-nAChR sequence. Individual datapoints in grey with loess-smoothed average in black.

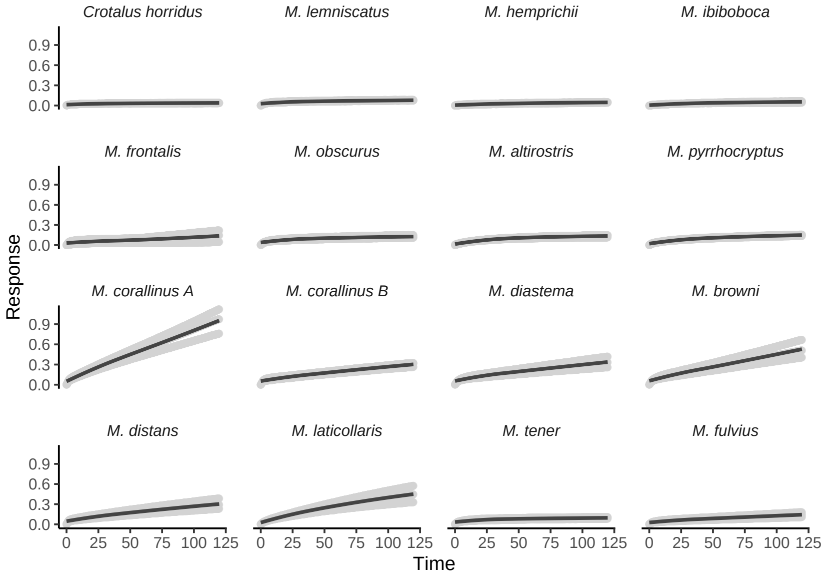

Figure S5: Bio-layer interferometry results for *Micrurus* venoms binding to a mimotope based on rodent (*Gallus gallus*, UniProt E1BT92) α_1-nAChR sequence. Individual datapoints in grey with loess-smoothed average in black.

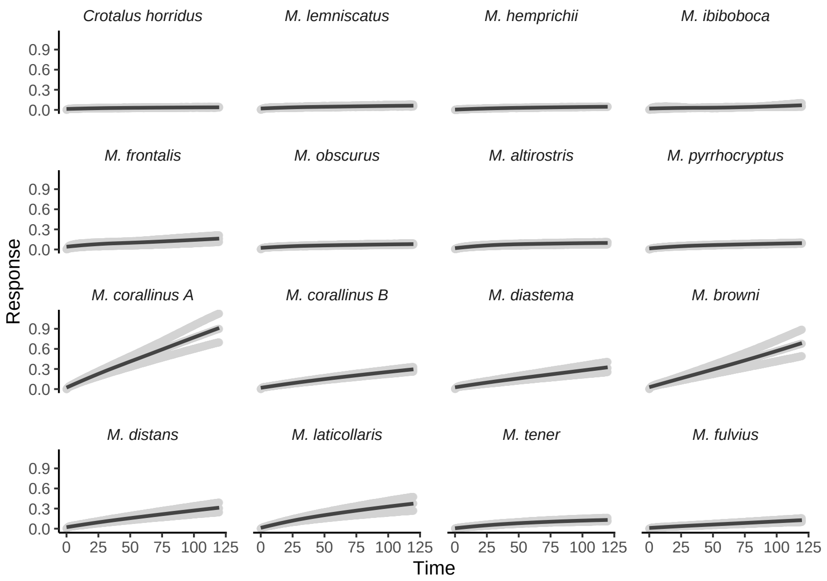

Figure S6: Bio-layer interferometry results for *Micrurus* venoms binding to a mimotope based on gecko (*Gekko japonicus*, GenBank XM015426640) α_1-nAChR sequence. Individual datapoints in grey with loess-smoothed average in black.

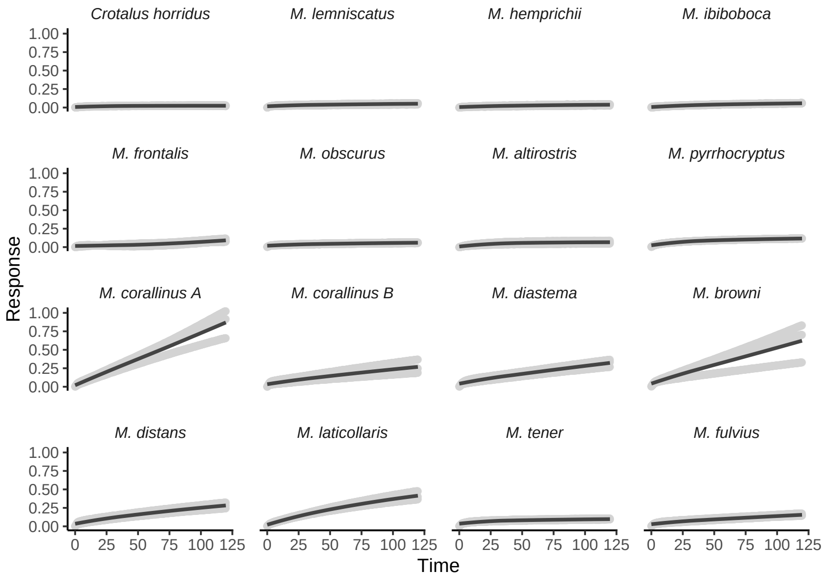

Figure S7: Bio-layer interferometry results for *Micrurus* venoms binding to a mimotope based on anole (*Anolis carolinensis*, UniProt H9GA55) α_1-nAChR sequence. Individual datapoints in grey with loess-smoothed average in black.

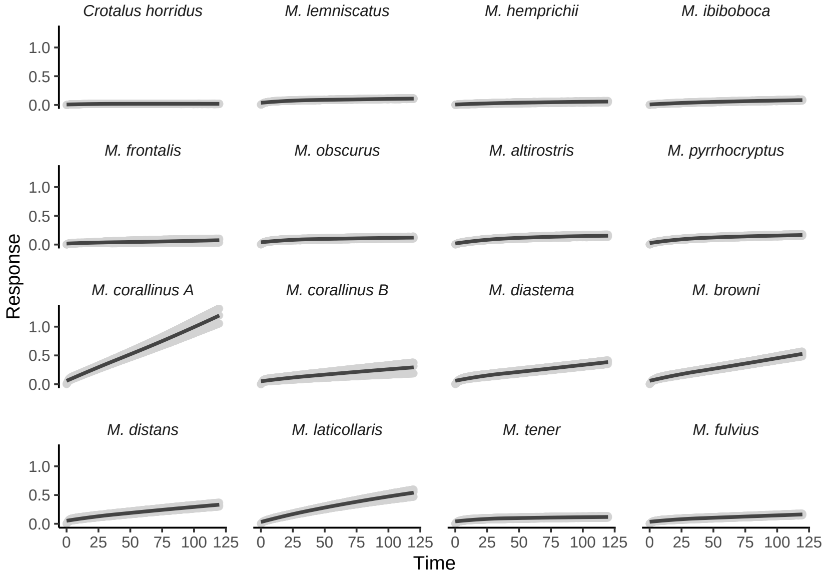

Figure S8: Bio-layer interferometry results for *Micrurus* venoms binding to a mimotope based on alligator lizard (*Barisia imbricata*, UniProt A0A859JD35) α_1-nAChR sequence. Individual datapoints in grey with loess-smoothed average in black.

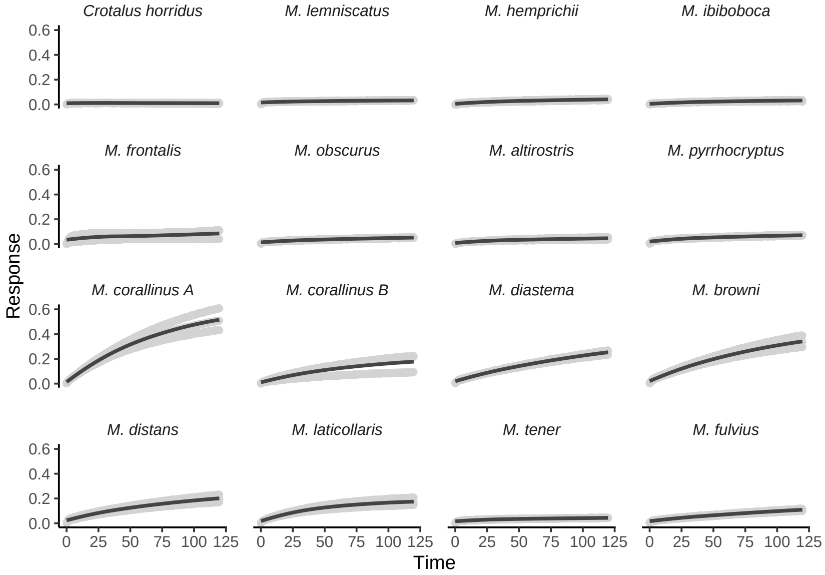

Figure S9: Bio-layer interferometry results for *Micrurus* venoms binding to a mimotope based on blindsnake (*Anilios bituberculatus*, UniProt A0A7L5PIU6) α_1-nAChR sequence. Individual datapoints in grey with loess-smoothed average in black.

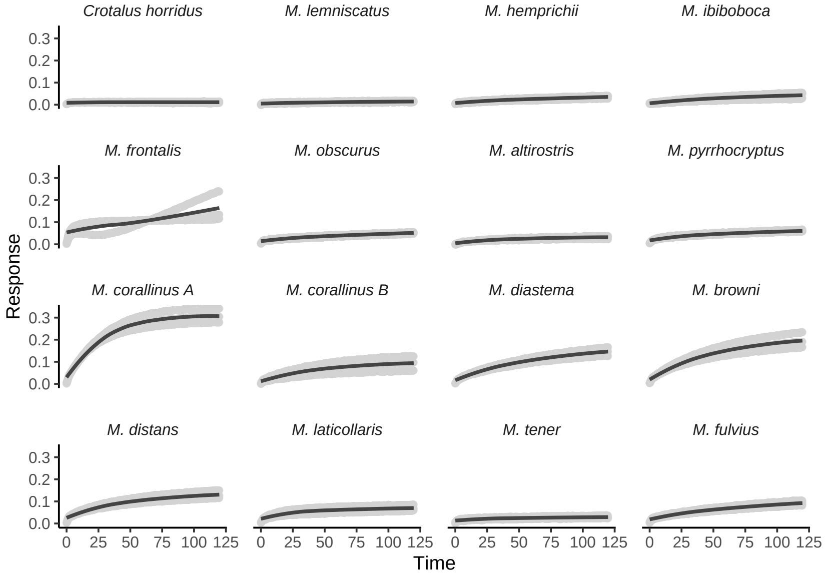

Figure S10: Bio-layer interferometry results for *Micrurus* venoms binding to a mimotope based on boa (*Boa constrictor*, UniProt A0A7L5PLV8) α_1-nAChR sequence. Individual datapoints in grey with loess-smoothed average in black.

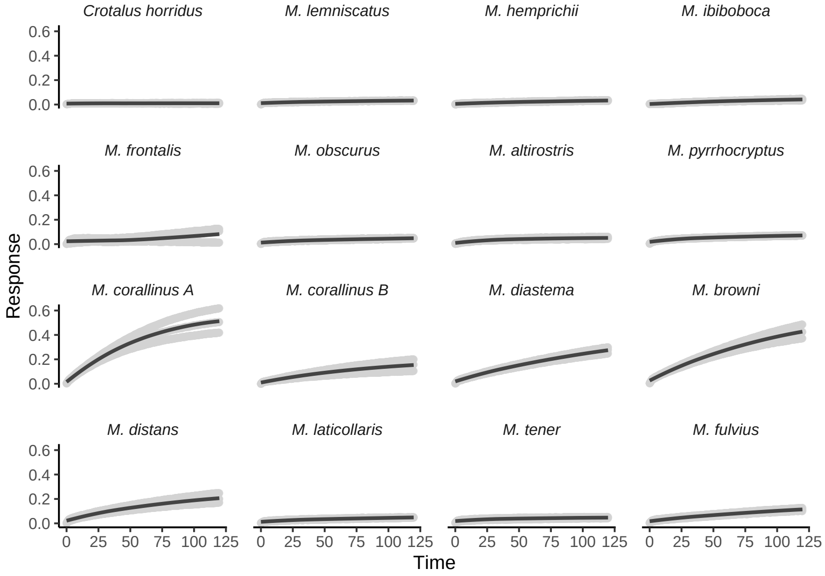

Figure S11: Bio-layer interferometry results for *Micrurus* venoms binding to a mimotope based on dipdsadine (*Oxyrhopus rhombifer*, UniProt A0A7L5PLX4) α_1-nAChR sequence. Individual datapoints in grey with loess-smoothed average in black.

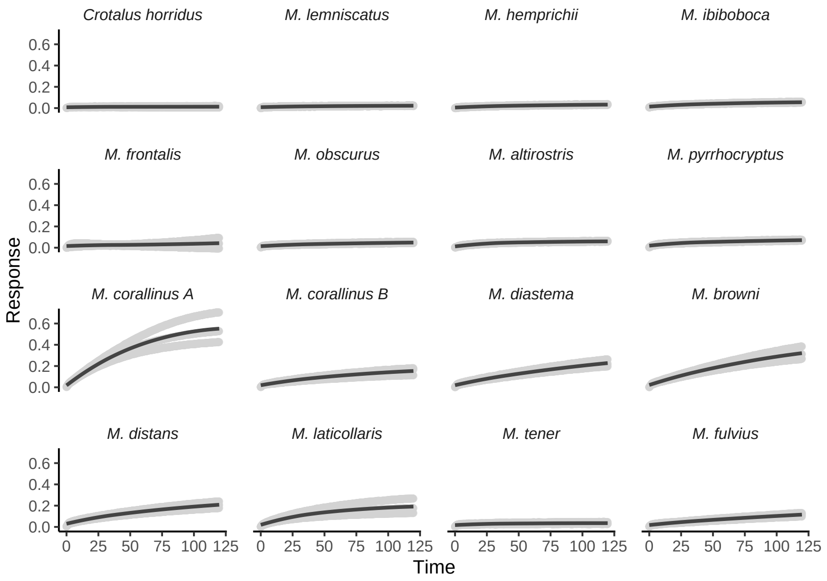

Figure S12: Bio-layer interferometry results for *Micrurus* venoms binding to a mimotope based on colubrine (*Pantherophis spiloides*, UniProt A0A7L5PL14) α_1-nAChR sequence. Individual datapoints in grey with loess-smoothed average in black.
